# Expressive Avatars: Vitality in Virtual Worlds

**DOI:** 10.1007/s13347-023-00628-5

**Published:** 2023-04-06

**Authors:** David Ekdahl, Lucy Osler

**Affiliations:** 1 Department of Sociology, Philosophy and Anthropology, University of Exeter, Byrne House, St German’s Road, Exeter, EX4 4PJ UK; 2 School of English, Communication and Philosophy, Cardiff University, John Percival Building, Colum Drive, Cardiff, CF10 3EU UK

**Keywords:** Avatars, Expressivity, Virtual worlds, Video games, Vitality

## Abstract

Critics have argued that human-controlled avatar interactions fail to facilitate the kinds of expressivity and social understanding afforded by our physical bodies. We identify three claims meant to justify the supposed expressive limits of avatar interactions compared to our physical interactions. First, “The Limited Expressivity Claim”: avatars have a more limited expressive range than our physical bodies. Second, “The Inputted Expressivity Claim”: any expressive avatarial behaviour must be deliberately inputted by the user. Third, “The Decoding Claim”: users must infer or figure out the expressive meaning of human-controlled avatars’ behaviour through cognitively onerous processes. With the aim of critically assessing all three claims, we analyze data collected through observations of and interviews with expert players of the avatar-based video game League of Legends. Focusing on Daniel Stern’s (2010) notion of vitality, we analyze the participants’ descriptions of seeing and interacting with other avatars during performance. Our analysis shows that the informants experience human-based avatarial interactions as qualitatively different than interactions with bots, that the informants see the movements of other players’ avatars as having different expressive styles, and that the informants actively use and manipulate this avatarial expressivity during performance. The results of our analysis, we argue, provide reasons for loosening or resisting the three claims concerning the limits of avatarial expressivity.

## Introduction

The concept of an ‘avatar’ derives its meaning from Sanskrit, literally referring to an earthly embodiment or incarnation of an otherworldly deity. As such, the concept has often been adopted to mean a concrete embodiment of something abstract. However, with the rise of digital technologies such as video games, ‘avatar’ is now typically understood to refer to a virtual image representing a particular person or entity in a digital space, often controlled by a human. In this way, the meaning of the concept has notably shifted from ‘something abstract made bodily’ to ‘something bodily made abstract.’

If we view avatars as an abstraction from or a stand in for a bodily human, it seems intuitive to suppose that any social interactions between human-controlled avatars will be similarly abstract, and, consequently, that interacting with another human-controlled avatar is removed or uprooted from our more concrete, everyday encounters with other people. To put it another way, when we meet one another mediated through avatars, we might expect that the way we encounter one another is significantly different to how we encounter one another when we are physically face-to-face.

While there has been significant work done on how people experience using avatars in virtual worlds, e.g., in terms of embodiment, presence, immersion, identification, self-identity, online friendships, and community (e.g., Badrinarayanan et al., 2014; Bülow & Felix, 2016; Crick, 2011; Ess, 2012; Farrow & Iacovides, 2014; Fong & Mar, 2015; Gies, 2008; Hardesty, 2016; Hilvoorde & Pot, 2016; Klevjer, 2012, 2022; Nilsson et al., 2002; Salinäs, 2002; Schectman, 2012; Schroeder, 2002; Schultze, 2010; Tartaglia, 2012; Taylor, 2002),^1^ there is comparatively little literature investigating our ability to express ourselves and understand each other through avatar interactions. Where the social implications of avatars are considered, it is commonly supposed that not only are our social encounters significantly altered when conducted through avatars but that the use of avatars hampers our ability to understand one another. The concern is that the expressive behaviour that is the hallmark of our bodily encounters, and crucial for our social understanding, is severely lacking, even absent, when we use avatars.

We can broadly identify three claims made about avatarial expressivity. First, that most avatars have extremely limited options to express emotions, actions, and intentions, as avatar bodies can only make a limited range of pre-configured facial expressions, gestures, and other non-verbal body language compared to our physical bodies (e.g., Moore et al., 2007; Scriven, 2018; Svenaeus, 2021)—we call this the “Limited Expressivity Claim”. Second, that the expressive options that avatars have are often cumbersome for a player to enact as they must be deliberately and consciously inputted by the player (e.g., Dreyfus, 2008; Scriven, 2018)—we call this the “Inputted Expressivity Claim.” Third, when faced with someone’s avatar rather than their physical body, we must cognitively figure out or decode the signals the other is giving us through their avatar (e.g., Berger, 2020; Dreyfus, 2008)—we call this the “Decoding Claim.” Taken together, these claims might lead us to conclude that avatars simply “do not express feelings in the same way that live human (or animal) bodies do” (Svenaeus, 2021, 89), and that, consequently, it is difficult to really understand others when we encounter them in digital spaces mediated by avatars.

In this paper, we provide an in-depth analysis of how a set of skilled players of the popular, competitive game League of Legends (LoL) experience and interact with other players’ avatars in-game. Drawing from interviews with these expert practitioners, we find evidence to loosen the Limited Expressivity Claim and resist the Inputted Expressivity Claim. We suggest that these two claims overlook a key form of expressivity of human (and animal) bodies—“vitality.” In his book *Forms of Vitality* (2010), Daniel Stern calls attention to the way in which we perceive the dynamic style of people’s movements and that perceiving vitality is crucial for our understanding of others. We suggest that the LoL players’ reports give evidence that they perceive the other players’ avatars as having vitality and that being sensitive to vitality gives them important insight into the other human players’ feelings, intentions, and experiences. Crucially, this expressive vitality is not (at least typically) explicitly inputted into the avatar by the player but arises through play. Moreover, the reports also put the Decoding Claim under pressure, as the players report *seeing expressivity* in the movements and actions of the avatars, rather than having to ‘figure out’ what the other players are feeling and experiencing.

By focusing on the accounts of LoL practitioners, we are notably analysing descriptions of experiencing and interacting with a category of avatars that, in their design, do not strive to be *photorealistic* as they are not, e.g., seeking to accurately mirror the subtle, expressive nuances of the user’s physical face or body. In other words, our analysis is not aimed at the increasingly photorealistic avatars that have been emerging over the past few years, especially in consumer VR tech (see, e.g., Meta, 2022), which, one might well argue, are also putting growing pressure on the above three claims about the limits of avatarial expressivity. Our analysis focuses on non-photorealistic avatars, like those of LoL, which continue to be widely used today in conventional forms of computing, whether for gaming, work, or socializing. In this context, it is notably these kinds of non-photorealistic avatars against which critics have primarily levelled the above, critical claims. Thus, rather than focusing on how, with sufficient technological advancement, photorealistic avatars can eventually be properly expressive, our analysis of the players’ reports explore how even non-photorealistic avatars have had expressive potential all along.

In Section 2, we outline the role that bodily expressivity plays in social understanding when we encounter others we are physically co-present with and we introduce the concept of vitality. In Section 3, we outline three claims about avatarial expressivity found in current literature. In Section 4, we introduce the game League of Legions and its avatars. In Section 5, we set out a brief overview of the methodology for obtaining and analyzing LoL players’ descriptions of their experiences with their own and other players' avatars in-game. In Section 6, we present three key themes that we identified in the LoL players’ reports: (i) humanity in the interaction, (ii) seeing ‘how’ players move, and (iii) using and manipulating expressivity during play. In Section 7, we return to the three avatarial expressivity claims in light of the LoL players’ reports. We argue that the LoL players’ descriptions put all three claims under pressure. By recognizing the expressive vitality of avatars, we argue that a greater degree of social understanding is possible when we encounter others in virtual worlds mediated by avatars than has previously been recognized. We conclude by considering several issues raised by our results and pointing towards suggestive future research.

## Encountering Expressive Others

### Bodily Expressivity and Social Understanding

Bodily expressivity plays an important role in our social understanding. When we encounter people face-to-face, we do not encounter identical mannequin-like bodies but bodies spilling over with expressive behaviour. We see happy smiles, worried frowns, concerned looks, mirthful glances, anxious postures, reserved behavior, confident strides, welcoming open arms, and so on. We see bodily movements and gestures as expressive of emotions, intentionally motivated action, personality, and unintentional reactions. Having access to other people’s bodily expressivity plays an important role in our understanding of others. Being able to perceive someone’s bodily expressivity helps us understand what a person is thinking, feeling, and doing.

Different social cognition theories grant different roles to the perception of bodily expressivity. For instance, *Theory Theory* (e.g., Gopnik & Wellman, 1994) suggests that when we see expressive bodily behavior we use this to form theories about what the other is experiencing and deploy these theories when we see similar expressive behaviour again. In this way, if Daisy sees Anna smiling, she calls upon her theory that ‘when people smile, they are happy’ to infer that Anna is happy at this moment. *Simulation Theory* (e.g., Goldman, 2006), on the other hand, suggests that when Daisy sees Anna smiling, she simulates what it would be like to be smiling herself and this gives her insight into Anna’s current experience.

We are sympathetic to the *Direct Social Perception* model of social cognition that argues that when Daisy sees Anna smile, she does not need to theorise nor simulate what Anna is feeling, she directly sees Anna’s happiness in their smile (e.g., Gallagher, 2008; Krueger & Overgaard, 2012). As Shaun Gallagher (2008) puts it, our social perception of others is a *smart* perception—we do not see muscles pulling the edges of a mouth upwards, rather we see this expressive behaviour *as* a happy smile. Our sympathy for *Direct Social Perception* is, in part, supported by the fact that many current articulations of *Theory Theory* and *Simulation Theory* now state that theorising or simulating often occurs at a subconscious level and, as such, all three theories appear to be in agreement that we often *experience* our understanding of expressive behaviour as direct, immediate, and non-inferential (see Spaulding, 2015).

The Direct Social Perception model of social cognition is often adopted and expanded upon in *enactivist* approaches, which stress the role of the affective, bodily behaviour of the participating interlocutors in driving social understanding (De Jaegher, 2009; De Jaegher & Di Paolo, 2007; Fuchs & De Jaegher, 2009). On such a framework, social understanding is not only experienced through the *perception* of the expressive behavior of other people but *emerges* through bodily interaction, attunement, and resonance between social participants. Not only are we sympathetic to these elements of enactive approaches to bodily expressivity and social understanding, we also consider these approaches resonant with the theoretical framework we introduce and deploy in our later analysis.

Irrespective of the specific social cognition theory, expressive behavior is given a central role in our everyday understanding of other people. Take away our access to the bodily expressivity of others and we take away a key feature of how we understand others.

### Perceiving Vitality

Discussions of perceiving bodily expressivity primarily focus on the perception of episodic emotions in discrete gestures. We find many examples of seeing someone’s happy smile or their angry frown. There is, though, a risk of describing bodily expressivity in overly static terms, where we only perceive isolated gestures or actions. This focus on discrete facial expressions has been criticised for overlooking the significance of the concrete context in relation to social understanding, thus failing to capture the importance of situation to our perception of others’ emotions and moods (Crippen, 2021; Crippen & Rolla, 2022).

By focusing on individual gestures, what falls into the background is a more holistic way of experiencing the other’s bodily expressivity. Stern, in his book *Forms of Vitality* (2010), argues that we do not simply perceive others in terms of *what* they do but the *how* of their actions and movements, their manner and style (2010, 4). The same type of gesture can have a different style when expressed by different people in different circumstances; for instance, we might perceive someone waving shyly, exuberantly, weakly, tentatively. What Stern is drawing attention to here is the dynamics of our expressivity, what he calls “forms of vitality.”

Stern claims that nearly all actions are marked by a vitality contour—a distinctive style of movement—and that our experience of vitality is “fundamental and primary” (2010, 25). He describes vitality as a gestalt that emerges from five interlinked dynamics: “movement, time, force, space, and intention/directionality” (2010, 4). Vitality is not an emotion but the tone of the dynamic flow of action and patterns of movement. Importantly, Stern does not see vitality as some kind of additional flair or detail that actions might have. Rather, he argues for the primacy of vitality: “The experience of vitality is inherent in the act of movement” (2010, 9). Stern suggests that it is vitality that marks the aliveness and dynamism of human action, stating that “[w]ithout manifestations of vitality, the world would be bereft of much of its interest, and human interactions would be digital rather than analogic, whatever that might be like” (2010, 4).^2^

An individual’s vitality is not fixed. The same action can be performed with different styles depending, for instance, on the context, their mood, their well-being; we might wave openly to a friend and furtively to a crush. Moreover, the vitality of an action can change over time. Joel Krueger (2021) illustrates this through the example of learning to play the guitar. When first starting out, an individual might strum a chord with uncertainty. However, as the player improves, striking the same chord may be done confidently. Thus, one’s vitality can betray how skilled one is at a particular action.

Stern’s notion of vitality captures an important way in which bodily movement is expressive and emphasises the place vitality plays in our experience of others. Important for our purposes, recognizing our ability to perceive vitality broadens the range of behavior that we might perceive as expressive of another’s subjectivity and experience (also see (Liu et al., 2022)). While Stern discusses vitality as something that we perceive as directly expressive and meaningful, and as such looks to be commensurate with a direct social perception and enactivist approaches to social cognition, note that one could retain a *Theory Theory* or *Simulation Theory* account of social cognition and still admit the perception of vitality as an important form of bodily expressivity that aids our understanding of others.

## Three Claims About Avatarial Expressivity

While social cognition literature has typically focused on how we encounter others when we are physically face-to-face with one another, there is growing consideration of how we might perceive the bodily expressivity of others when we encounter them online (e.g., Ferencz-Flatz, 2022; Jackson, 2021; Osler, 2021; Osler & Zahavi, 2022; Svenaeus, 2021; Vidolov, 2022). For instance, Lucy Osler (2021) argues that when we use digital platforms such as Zoom, FaceTime, or Skype, although bodily expressivity is mediated by screens and speakers, we still have perceptual access to others’ expressive behavior. However, we might be concerned that when we encounter others via other digital mediums, such as email or instant messaging, that we lose access to the bodily expressivity of others, and this leaves us with less information to understand them. As Thomas Fuchs (2014) puts it, when we go online, we engage more in disembodied communication via written signs and symbols, rather than encountering others in their embodied presence. As such, we are limited to encountering linguistic expressivity and no longer encounter rich bodily expressivity. Fuchs (2014, 167) argues that this “non-sensuous means of communication leave[s] so many blank spaces,” which we must fill with guesses, projections, and imaginings.

What, though, about when we encounter others not through written signs and symbols, but mediated through an avatarial body? On the face of it, it seems obvious that encountering an avatar is not the same as encountering someone face-to-face. Rather than being able to see what someone is doing and feeling, we are faced with a digital figure—a construct or image that may have little to no resemblance to the player’s actual bodily form and whose actions do not necessarily correspond to the actions that the player’s physical body is making. Unlike with the case of a physically co-present encounter, or even a livestreamed video call, we cannot see someone’s bodily expressivity. We see an animated picture and what is available to us is whatever the avatars themselves can be made to display.

Critics have, in this context, insisted that avatars simply fail to provide us with the kind of expressivity that facilitates the nuanced social understanding that we can gain of others in the offline world. Even those who want to allow for the possibility of rich social understanding in certain digitally mediated contexts often draw the line when it comes to avatars. Frederik Svenaeus (2021), for example, argues that, while we might perceive the expressive behaviour of others on video calls, we should not extend this analysis to the case of avatars:

Avatars, used in multiplayer on-line games, which you watch on the screen as you move and take action from your own first-person perspective, do not even come close to the experiences you will have if you, say, play hide and seek in a wood together with friends. Avatars in popular games played on PCs, consoles or mobile phones often resemble human bodies, but they do not express feelings in the same way that live human (or animal) bodies do (Svenaeus, 2021, 89).

What Svenaeus emphasizes is not merely that there are qualitative differences between human and avatar interactions—something relatively uncontroversial—but that the latter lack the kind of expressivity required for grounding social understanding; that seeing and interacting with and through avatars is socially too “thin and restricted” (Svenaeus, 2021, 89) to provide us with an understanding of the player ‘behind’ the avatar.

The worry is, then, that far from enriching the kinds of social encounters and interactions that we can have in digital environments, avatars ‘get in the way of’ our grasp of and understanding of others. We have identified three related but separable claims about avatarial expressivity and how this hampers social understanding in the literature on avatars that we dub *The Limited Expressivity Claim*, *The Inputted Expressivity Claim*, and *The Decoding Claim*.

### The Limited Expressivity Claim

Current accounts of avatarial expressivity often remark on the limited and constrained options available for players to express themselves. In a game such as LoL, for example, while players can move their avatars around the digital world, interact with digital objects and environments, and interact with other player and non-player characters, there are few options for making one’s avatars perform expressive gestures and actions. Indeed, in many games, players rarely even see other avatars ‘up close’—often experiencing other players from an overhead bird’s eye view. A common concern, then, is that avatarial bodies “give off” far fewer cues about what users are doing than real human bodies” (Moore et al., 2007, 22; also see Berger, 2020). Rather than the minute and constant expressivity of human bodies, avatarial bodies appear relatively static.

As Paul Scriven puts it, the concern is that when it comes to avatars: “more or less gone are unconscious body language, state of consciousness, facial cues, and so forth” (Scriven, 2018, 203). When compared to the array of expressive movements that our physical bodies make, avatars seem like a very poor substitute (Turkle, 2011). The paucity of options that players have for expressing themselves through avatars in online games like LoL is often highlighted. This we call the “Limited Expressivity Claim.” If avatars lack, or have very limited, possibilities for making expressive gestures and movements, it seems that any expressive behaviour present for other players (or spectators) to perceive is also lacking.

### The Inputted Expressivity Claim

In a related vein, we find discussions that emphasise not *what* expressions an avatarial body is able to make (or not make) but the *way* in which players express themselves through their avatars. Hubert Dreyfus (2008, 113), commenting on the online, avatar-based platform Second Life, draws a sharp distinction between how bodily expressivity unfolds in face-to-face encounters and the expressivity of avatars as follows:

...in the real world our bodies *spontaneously* express our moods and others…while in *Second Life* one has *to select* an appropriate gesture and then *command* one’s avatar to make that movement…

His concern is not simply that an avatar’s range of expressive options is limited but that the very way in which players express themselves when using avatars is different to our everyday bodily expressivity. While we usually do not make conscious decisions about how and when to express ourselves bodily, Dreyfus argues that when it comes to avatars the player must decide when and how to make expressive gestures in game. Scriven (2018, 203) also echoes this claim:

Expressions by the player character *necessarily* require input from the controlling player. The player must make decisions as to where the player should go, how the player character should interact with the environment in a way that meets the player’s goals, and, where relevant, how to communicate with others via textual or voice chat.

What both Dreyfus and Scriven suggest is that, when talking about avatarial expressivity, we are at a far remove from the everyday world of instantaneous, pre-reflective or even unconscious bodily expressivity. Instead, players must cognitively, consciously, and deliberately decide what to signal to others in an indirect manner. Thus, avatarial expressivity is described as emerging from deliberate cognitive choices and marries up with Jim Parry’s description of online gamers as “distanced, image-manipulating remote-controllers” (2018, 10). This we call the “Inputted Expressivity Claim.”

Note that we can, of course, deliberately perform bodily expressive gestures. One might wave exaggeratedly to get someone’s attention, make a show of nodding along to someone’s talk to indicate attentiveness, and mask one’s annoyance with a smile. To clarify, the claim made by Dreyfus and Scriven is not that we never consciously decide how to move our physical bodies in expressive ways but that our physical bodies are also sites of expressivity where smiles, frowns, hesitations, nose wrinkles, widened and narrowed eyes often play out without deliberation but when it comes to avatars we are limited to only expressivity that is explicitly inputted and performed by the player. As such, to the extent that we want to allow that avatars’ movements can be expressive of the player’s intentions and emotions, this expressivity is still notably different from the lively immediacy of bodily expressivity.

### The Decoding Claim

The final claim moves from discussing the expressive range of avatars and the way in which players can express themselves through avatars to a claim about how players view the avatars of others. Returning to Dreyfus, we find the claim that while we “*directly* pick…up” emotions, moods, and intentions of others when we encounter them face-to-face, and perceive fleeting smiles, furrowed brows, and sceptical looks as immediately meaningful, when we encounter someone’s avatar, we must “*figure out* what the [avatarial] gesture means” (Dreyfus, 2008, 113). In a similar vein, Viktor Berger (2020, 606, our italics) writes:

In contrast to the abundance of information of physical co-presence, each player has to *draw conclusions* about the inner consciousness of others using a limited set of perceptions: player-driven actions, programmed emotional reactions and the static movements of their avatar, as well as textual and voice-based communications.

According to such views, when we encounter others mediated by avatars, we are shouldered with the cognitive burden of trying to decode what the movements of their avatar could possibly mean or signal to us. To use the terminology of direct social perception, we might say that face-to-face our social perception is *smart* but avatars work to disrupt or prevent this smart perception. As such, when we interact via avatars, we must fall back on cognitively onerous means of working out what other people are thinking and feeling.

Note again that this is not a claim that we always see bodily expressivity as immediately meaningful and clear. Indeed, bodily expressive behaviour can often be ambiguous (Gallagher, 2008)—think of how we might be unsure whether someone is frowning in concentration or in disagreement during our presentation or whether someone meant to give the newcomer the cold shoulder or simply has not yet noticed them enter the room. Rather, it is the claim that often we perceive bodily expressivity as meaningful directly but when it comes to avatars this immediacy evaporates and cognitively working out what avatarial movements might mean is our only option.

### The Three Claims

While the *Limited Expressivity Claim*, the *Inputted Expressivity Claim*, and the *Decoding Claim* are often run together, it is important to recognize that the claims are different. The *Limited Expressivity Claim* comments on the *range of the expressiveness of avatarial bodies*; the *Inputted Expressivity Claim* suggests that even if the available expressive options were greater, there is *something fundamentally different about how we express ourselves through avatars compared to our everyday bodily expressivity*; and the *Decoding Claim* argues that unlike our immediate grasp of bodily expressivity, we must *decode the movements of avatars to work out what the player behind the avatar is thinking and feeling*. What are the implications of these concerns? Taken together, these claims seem like three nails in the coffin for the idea that we can gain good social understanding of others when we encounter them mediated by avatars. Platforms that use avatars, be it gaming worlds or social platforms, may look like they give us the possibility to interact with others via digital bodies but these claims suggest that they do not do a good job of replicating our social interactions carried out face-to-face. This, then, likely leaves us sceptical about the possibilities that such platforms provide for rich social interaction and understanding.

It should be noted that it is not clear whether the purported qualitative difference between avatar-based expressivity and bodily expressivity is considered to be a universal or an empirical constraint. As Dreyfus (2008, 119) points out, the supposed deficiencies in relation to avatarial expressivity might well be a limitation of current technology and not a universal constraint on avatars per se. Regardless, the fundamental claim of the critics mentioned above is that, as of today, avatars broadly lack the kind of expressivity required for meaningful social understanding. The aim of our paper is to scrutinize the three claims about avatarial expressivity and social understanding identified in this section in relation to currently available andconventionally used forms of technology.^3^

To do this, we analyze how skilled gamers report seeing and interacting with avatars when playing LoL, a game played by millions of players around the world every day. Before turning to the themes arising from our analysis of the LoL players’ descriptions, as well as our discussion of avatarial expressivity, we first provide some background about LoL and outline our methodology.

## League of Legends and Its Avatars

Avatars come in many different forms, with varying aesthetics and ways in which players can control and move them. When discussing avatarial expressivity, it is important to be specific about what platform is being used, as the possible actions one can carry out through one’s avatar depend on the design of the platform. Here, we consider avatarial expressivity in the context of modern video games, specifically how skilled players of LoL experience and use avatars in game.

LoL is a real-time, multiplayer online battle arena (“MOBA”) game built around various genres of high fantasy, steampunk, and sci-fi. The game’s standard competitive format pits two teams of five players against each other, each player in control of an avatar with a unique set of abilities (known in-game as a “champion”),^4^ moving and coordinating with their team in real-time across the virtual map.^5^ The game takes place from a bird’s eye view, with each player able to move their own ‘lens’ or ‘camera’ on the game world around freely to scout the map around them. In this way, the entire game plays out from a top-down perspective and at no point does the player view the game world ‘through’ the eyes of their avatar as in first-person perspectival video games.

The avatars themselves are fantastical, ranging from yeti-looking creatures and minotaurs to tiny furry beings known as “yordles.”^6^ Each avatar comes with several “skins”—graphical redesigns of varying rarity that players can choose when selecting their champion. Each champion has a unique set of abilities, typically designed for excelling at a particular role on the team, such as dealing damage or protecting one’s teammates. For this reason, a skilled player usually specializes in a particular role and only a handful of different champions.

From a gaming perspective, movement in LoL is paradigmatic of many video games. Players move their champion around in-game primarily via clicks of their mouse. By right-clicking somewhere in the game world, their avatar will move to the clicked location unless stopped, e.g., by other in-game forces such as other players’ avatars, or by being redirected, e.g., by giving the avatar a new command through the click of their mouse. During performance, players will often execute several hundreds of movement commands every minute, primarily in order to adjust their avatar’s current position, as well as to deploy its abilities. In general, the only kinds of avatar actions available to the players are the mouse-directed relocation commands of the avatar (the avatar’s standard movement), as well as the aiming and use of the avatar’s abilities, which typically occur through a combination of keyboard and mouse use. While so-called “emotes” exist in LoL, which can cause one’s avatar to dance or laugh, these are rarely used once a game begins.

In terms of communication, players can write to each other using the in-game text-based chat, even across the two teams if they wish, as well as via an in-game “pinging” system, used to immediately draw teammates’ attention to specific areas or events on the game map. In organized and competitive LoL, players typically communicate verbally with their own team using headsets.

## Method

The data used in this paper are part of a larger set generated in the years 2018–2019 by the first author in connection with a research project on the phenomenology of organized competitive videogaming—also known as ‘esports’ (also see Ekdahl, 2021, 2022; Ekdahl & Ravn, 2019, 2022). The subset of data we analyse here is based on ten semi-structured interviews with ten Danish esports professionals (see Table 1 for an overview of the informants)—supplemented by earlier periods of observations of the informants’ training sessions or pre-interview meetings with the informants if observations were not possible. Both pre-interview meetings and periods of observation served to contextualize the interview situation as well as hone the topics and questions of the interviews. Each interview lasted between one to two hours, with an average interview length of around ninety minutes. The transcribed interviews included response tokens, diction, non-verbal vocalizations, and game-specific slang. The transcribed interviews excluded geo-ethnic accents, involuntary vocalizations, and personal pronunciation (Oliver, et al., 2005). The interviews were translated from Danish to English by the first author.

**Table 1:** The informants

*Pseudonym*	*Participant background with gaming/esports*
Lyn	LoL trainer at a boarding school for lower secondary students. Former semi-professional LoL player. Top 0.28% ranking in LoL
Balder	LoL trainer at a public school. Top 1.4% amateur ranking in LoL
Liam	LoL trainer at a high school. Top 0.14% ranking in LoL
Ben	LoL trainer at a boarding school for lower secondary students. Top 6.6% ranking in LoL
Brett	Free-lance LoL trainer. Top 2.1% ranking in LoL
Lucas	Esports psychologist. Top 14% ranking in LoL
Loke	Esports head coach. Top 1.3% ranking in LoL
Leif	LoL coach. Top 0.1% ranking in LoL
Leon	LoL coach. Top 0.1% ranking in LoL
Brian	LoL trainer. Top 1.0% ranking in LoL

The study’s approach was inspired by the qualitative-phenomenological methodology of Ravn (2023, see also Legrand & Ravn, 2009) and the two-tier, phenomenological interview model developed by Høffding and Martiny (2016). Focusing especially on the LoL practitioners’ descriptions of seeing and interacting with other avatars, the data was first analysed exploratively (Ravn, 2023) with the aim of identifying central, emic themes (Hammersley & Atkinson, 2007) through an iterative process where the interviews were read through until internal consistency could be established. These themes included ‘tilting,’ ‘bots versus humans,’ ‘jittering,’ ‘temperament,’ ‘seeing avatars move,’ and ‘bluffing/feinting.’ This led to our second-tier analysis of the LoL practitioners’ descriptions, here with a focus on Stern’s (2010) framework of vitality forms. It is this analysis that proceeds in Section 6.

Our analytic strategy can be said to follow the case study methodology described by Flyvbjerg (2011). A strength of this approach, as noted by Schiavio and Høffding (2015, 6–7), lies in its ability to produce both petite and grand level generalizations – something also discussed by Stake (1995, 7). Whereas petite generalizations can be described as a “within-case” generalization, e.g., case-specific themes identified across the informants’ descriptions, grand generalizations refer to a larger scope of generalization beyond the case itself, for instance, with reference to ideas held by the broader academic landscape (Ravn & Christensen, 2014; Stake, 1995). In this way, our analysis not only develops our understanding of a specific subset of avatar interactions, it also aims to engage critically with the broader body of literature on avatarial expressivity, and, in doing so, open up novel research questions in this field (Flyvbjerg, 2011, 343).

## Vitality in LoL

Here, we present our analysis of the practitioners’ experiences of avatarial interaction in LoL. Our analysis comprises three parts: (i) encountering player avatars vs. bot avatars, (ii) perceiving the vitality of other players’ avatars, and (iii) using and manipulating vitality in-game. Based on this analysis, we move away from characterising avatars as essentially lacking expressivity and instead highlight the sensitivity that practitioners have to the forms of vitality that players express through their avatars and the role this plays in social understanding and performance in-game.

### Humanity in the Interaction

When examining avatarial expressivity, a good place to start is comparing the players’ experiences of human-controlled avatars with their experiences of computer-controlled avatars or ‘bots.’ If we suspect that avatars lack the kind of expressivity that physical bodies have, then we might also suppose that players would be unable to differentiate between human-controlled avatars and bots. In LoL (and similar games of the same genre), the standard bots do move and act in a manner where a complete LoL novice might, at first, be somewhat hard-pressed to tell the difference between the bot and a human player. An enemy bot seemingly reacts to the activity of the player in relatively responsive ways, withdrawing when in danger and pushing forward when the player is in danger. However, with just a little practice, the differences between a human and a bot become obvious. During an interview, the distinction between bots compared to human-controlled avatars is especially emphasized by LoL professional Lyn:

Interviewer: [can one] provide a meaningful difference in the way one experiences a bot compared to the way one experiences a human player given that one does not have direct access to the other actual human? ...

Lyn: That’s the thing. Because when I compare this, then I think back on the actual bots you can play against. They move…*almost* human-like, but they do not move in quite the same *manner* [Lyn’s emphasis] (Lyn Interview 2, lines 867–869, 871–872).

For Lyn, there is something subtly but qualitatively different between encountering a human player and a bot. Indeed, the very idea that a bot *could* present in the very same manner as a human-controlled player elicits the following response: “I would be very surprised, I would be befuddled…” (Lyn Interview 2, line 865).

Being able to differentiate a bot from a player impacts how a player engages with an opposing avatar. LoL professional Lucas specifically elaborates on this, describing that beating a standard bot in LoL involves using one’s avatar in a calculative manner—for instance, by making mistakes on purpose to try and gauge the bot’s predetermined responses (Lucas Interview, lines 429–431). In contrast, when facing skilled players, Lyn describes how these are normally experienced as more unpredictable, moving in complex and irregular patterns (Lyn Interview 1, lines 890–898).

One might surmise, then, that the difference between fighting bots and humans can be chalked up to the complexity and unpredictability of beating other human players compared to bots. While unpredictability is a central feature of human avatarial interactions, focusing on this alone as the central distinctive feature contradicts the LoL informants’ descriptions of fighting more challenging computer-controlled opponents available. As an example, in LoL, players can battle extremely capable bots – so-called ‘Doom Bots’, computer-controlled avatars that spontaneously and unpredictably acquire high-powered and novel abilities throughout a match. Yet, even in this case, “…there is little humanity *in* the interaction”, Lyn explains, emphasizing that, despite their complexity and unpredictability, she does not experience these bots as making any real decisions, but rather as her *causing* the bots to act in certain ways based on her own avatarial input [Lyn’s emphasis] (Lyn Interview 2, lines 876–878).^7^

Based on these descriptions, we can appreciate that human-controlled avatarial movements and actions can be characterized by something beyond the unpredictability and skilfulness of the interactors themselves—that there seems to be something distinctly human about fighting human-controlled avatars. In contrast to Lyn’s descriptions of merely *causing* bots to respond to her own actions, the practitioners do not appear to experience other player-controlled avatars as moving around and acting in an automatic, predestined manner. In drawing attention to the idea of humanity *in* the interaction, Lyn appears to point to not simply what bots or player-controlled avatars are *doing.* Rather, she highlights the way in which player-controlled avatars are not simply responsive to actions but are experienced as an agent *in* the interaction, as some kind of reciprocal other.

### Seeing ‘How’ Players Move

Let us now turn to how practitioners describe encountering human-controlled avatars in more detail. In LoL, opposing players' avatars will often ‘jitter’ rapidly around each other in a manner as a product of their hundreds of rapid but precise clicks per minute on their gaming mouse to relocate their avatar in the virtual space. An important reason for jittering like this, according to the informants, is to make themselves a more unpredictable and difficult target.

When several human-controlled avatars are jittering near each other, they come to constitute what Lyn describes as an in-game “dance” (Lyn Interview 1, lines 927, 931, 967–971)—a dynamical back-and-forth of trying to step close enough to or angle your position in an optimal way to hit and pressure your opponent, while dodging and weaving to avoid potential, retaliatory attacks. The resulting interaction will likely look like a fast-paced and pixelated mess to a LoL-outsider, increasing in complexity the more avatars are jittering around near each other simultaneously. In-game, from a strategic standpoint, jittering is an effective way to move against human opponents, but, importantly for our purposes, these interactions between human players are also described as animated by the practitioners in ways that bot interactions are not (Lyn Interview 1, lines 890–989; Balder Interview, lines 168–172; Liam Interview, lines 361–370; Loke Interview, lines 264–270).

We might then be tempted to think that the distinction between human-controlled avatars and bots rests on the presence of jittering; that jittering is what makes human-controlled avatars human-like in the way bots are not. However, bots can also jitter. Is there, then, a single distinctive manner in which human-controlled avatars move that make them, as Lyn describes it, “human-like”? The answer to this seems to be no. Practitioners are quick to emphasise that human-controlled avatars do not all move in one uniform manner. For instance, even when different human players control graphically identical avatars, they each move and act in different manners. When asked if he would experience the same identical avatar played by different human players across different rounds in a match distinctly, Loke explains:

You would. It is something you just… experience, right? It’s this thing once more about *seeing* it. The way you become conscious of the human player is in the way they perform and the way they move [Loke’s emphasis] (Loke Interview, lines 312–315).

In this way, the informants’ descriptions indicate that there seems to be something about the *manner* that human-controlled avatars move and act that not only distinguishes them from bots, but, as Loke notes, also between individual players. That is to say, human-controlled avatars seem to have an expressive range that bots do not. Echoing Stern’s discussion of vitality forms, it is not just *what* human players are doing that is different, there is something about *how* they do it that is distinctive (even between one another). In addition, not only are the dynamics of the avatar central to Loke’s experience of the other avatar as a human player, but this experience is even described in terms of “seeing it.”

Lyn likewise emphasizes the importance of seeing and reading others’ avatarial “body language” (Lyn Interview 1, lines 624–626, also 787, 935, 976), stressing that, while she “knows it sounds dumb,” she can see how other players in-game are doing mentally based on how their avatar moves and acts (Lyn Interview 1, lines 624–626), and later even adding that it is actually “really difficult to hide one’s intention about what one is doing…” (Lyn Interview 1, lines 936–937). She elaborates when further describing the phenomenon of experiencing the body language of other avatars:

Depending on how quickly the avatar moves, you can see if the person is stressed or not. If they move rapidly with small clicks all over the place, this normally means the player is stressed. Whereas if it is this random, nice and easy clicking over all the time, then the person is calm and in control – and on top of the situation. So, there you can read it (Lyn Interview 1, lines 979–982).

Based on the flow of subtle stylistic differences in the patterns of movement between the jittering avatars, the informants notably use a wide repertoire of adjectives and adverbs to describe their immediate sense of how different human-controlled avatars move, including “pressured,” “brave,” “apathetic” or “frustrated,” “aggressive,” and “supportive” (Brian Interview, lines 416–429; Liam Interview, line 393; Leif Interview, lines 262, 957, 965–970). Loke notes that when consistently outplaying an opponent, he will “sense their frustration” through their avatar (Loke Interview, lines 357–364) and Lyn explains that when cooperating with a familiar teammate, she will be able to see if her teammate’s movements and playstyle seem especially “nervous” that day, and even what “general mental state” the teammate enters the game in (Lyn Interview 1, lines 628–630). Informants even describe being caught by surprise at how expressive an opponents’ movements can be, with LoL practitioner Leon noting that he will catch himself thinking “Wow, he is stressed—that guy!” (Leon Interview, lines 696–697). Note that the practitioners are not referring to discrete expressive gestures made by an avatar here (for instance smiles, frowns, clenched fists) but to the dynamic patterns of movements and actions of the avatar. The “body language” they are referring to is the holistic way the whole avatar of the human player is moving in a temporally extended manner; it is the tone of the dynamic flow of action and patterns of movement; it is the vitality contour of the player’s avatar. Experiencing player-controlled avatars as other *players*, rather than bots, then, seems to be underpinned by a sensitivity to the vitality contour expressed in the player’s playstyle. This suggests that the experience of another player’s autonomy and agency through their avatar is not simply linked to *what* they do but to the way in which their temperament, experiences, and emotions shine through in their movements and actions.

Moreover, while players often bring their own temperament and style of moving and acting to their gameplay, as Leif notes (Leif Interview, lines 296–311, 317–333), these can change over time during a match depending on the in-game circumstances. In response to a highly pressured in-game situation, the avatarial style of a player with an aggressive playstyle might grow and appear more cautious during a round, and vice versa. That is to say, their vitality is not fixed. Likewise, any player’s general style, like Krueger’s guitar player, will also develop from their time as novice to proficient.

Avatarial expressivity is notably not something the LoL players typically pay explicit attention to during performance – even when they are directly engaged with other players. As we have seen, the trainers describe “seeing” the others’ avatars as expressive. As noted by Loke, it is something that becomes an “intuitive feeling” (Interview Loke, line 384); a pre-reflective part of their experience of the other players’ in-game movements and actions. Nor, importantly, are these expressive forms of vitality typically directly inputted by the player into their avatarial actions. For instance, a player does not necessarily present as stressed because they are deliberately and intentionally inputting that expressive style into their avatar’s movement; rather, the movement of the avatar inherently *has* this vital aspect based on how the player is currently playing. Indeed, as we have seen, the player may be unaware that their stress is shining through in the way they are moving their avatar around. To put it another way, there is not a button or a menu for inputting vitality into these patterns of movement (cf. selecting a pre-programmed wave), the vitality emerges *through* the way the avatar dynamically moves and interacts with other avatars and the virtual world.

Through the unfolding, even changing, expressive style and vitality of an avatar’s movements and actions, the humanity of the other player seems to become salient for the LoL players. Moreover, seeing human-controlled avatars as expressive appears to impart social understanding of the player ‘behind’ the avatar within the context of the game world. However, this is not based on the perception of the static expressivity of discrete facial expressions, but on the perception of forms of vitality of the player’s avatar’s movements and actions in the game. By moving away from scrutinizing how avatars ‘emote’ or how they fail to replicate offline bodies, to looking at how players experience avatarial unfolding, dynamic movement as expressive and personal in terms of vitality, we are able to appreciate an important aspect of avatar expressivity, and a significant part of how players perceive each other during performance, that has so far been overlooked.

One possible interjection here is that whatever social understanding of an opposing player is imparted via avatarial expressivity merely amounts to instances of projection. That is to say, one might interject that players simply read their own, e.g., emotional states into the avatarial movements of other players. There are at least two reasons to be sceptical of this. For one, the practitioners by no means have to be undergoing the same emotional state that they perceive through the avatarial movements of other players. Second, the expressivity of one’s own and other players’ avatars, including the imparted social understanding, are not mere addendums to the experience of proficient LoL interactions. As we shall see, far from projecting, e.g., one’s own emotions onto other players’ avatars, the practitioners’ ability to accurately see and engage with an opponent’s state of mind can be integral to successful LoL performance.

### Using and Manipulating Avatarial Expressivity

The practitioners do not simply report being able to see the expressive forms of vitality in relation to human-controlled avatars. Being able to see these expressive forms of vitality *informs* the way that the practitioners play. One informant, Balder, concludes a longer description of how he experiences movements in LoL by noting:

There is something in these… movements, where you can read a whole lot. I guess that is how it is. I never thought about it like that, but that is really how it is. Right, right—that’s what one does! And it is actually from *that*, that one makes his decisions along the way! [Balder’s emphasis] (Balder Interview, lines 168–172)

Players not only report acting upon the expressive movements and actions of others’ avatars but also that they sometimes use their own expressive styles in a strategic manner. For instance, a player can exaggerate a particular playstyle in order to draw out or learn something about the style and even temperament of the opponent.

The way I often do it is… I usually—and this is one of my secrets—I usually play insanely aggressively right from the beginning in order to see “What do they do if I play insanely aggressively?” (Balder Interview, lines 119–122)

Adding to this, players sometimes use avatarial expressivity as ways to deceive their opponents. As an example, Balder later notes that “[i]f you are well ahead, but [the opponent] keeps playing very defensively, then you can underplay how strong you really are” (Balder Interview, lines 162–163). Manipulating one’s own avatarial style, such as moving, and acting meekly, as Lyn similarly describes, can work to draw an overly cautious opponent in (Lyn Interview 1, lines 936–944). Such a move not only involves a sensitivity to the other player’s expressive style but also involves deliberately performing a particular form of vitality, knowing that the other players will perceive one’s expressivity in a particular manner. In this regard, it is precisely because the avatarial expressivity of other LoL players ordinarily can impart accurate instances of social understanding, that being able to at times distort this imparted social understanding can be integral to successful performance, as it can be in offline, physical competitions too.

Adding further nuance to this, both in the context of *seeing* and of *using* avatarial expressivity, other in-game actions can also play a role, such as *attacking* and *using abilities*. While the practitioners do not describe any explicit experienced differences in expressivity between, e.g., avatarial movements and avatarial attacks and ability use, their descriptions do point to an expressive relation between these during performance. That is to say, attacking actions can factor into avatarial expressivity, with Lyn noting that, depending on whether or when an opponent attacks her when she moves about in a” tough” or “confident” manner, “flexing her muscles”, this can tell her a lot about the person she is fighting (Lyn Interview 2, lines 771–774). In addition, factors such as how far away an avatar is able to hit an opponent (its *attack range*), as well as its unique set of available abilities, can further factor into what an avatar’s movements express. For example, when and how a player controlling a melee-ranged avatar’s movements appears aggressive or cautious (whether deliberate or not) can be very different from when and how the movements of a longer-ranged avatar might do so—something a capable player remains sensitive to throughout their performance. Expressive style, then, is shaped by the overall in-game situation.

Recognising that players see and use expressivity as part of their in-game performance also sheds further light on a classic strategic move used in many forms of gaming: tilting. In games like LoL, tilting refers to the way that players either themselves experience or attempt to elicit a strong emotional reaction from an opponent that obstructs and disrupts their performance. In this context, if an opponent’s style quickly changes from confident to frustrated or frenetic in response to pressure in-game, players can tell that this opponent is further susceptible to becoming tilted, for instance by ganging up on them repeatedly throughout a match (even if the team has to invest a lot of time and resources to do so) in order to render their play ineffective long-term.

The expressive avatarial activity is not only something that the players see but is something they actively use and act upon as competitors. Being sensitive to the expressivity of the other players is not just an interesting detail about how avatars can be experienced in-game but can be imperative to successful performance. Where players deliberately obfuscate or manipulate the way they move to convey a particular expressive style, these tactics work because the player not only can see their opponent’s style but presumes that their opponent can also see their own expressive vital style.

## Expressive Avatars

As we have seen, there has been much scepticism about the extent to which avatars can, if at all, be experienced as expressing a player’s thoughts, feelings, and actions. As detailed above, this scepticism is often expressed in three related, but separable, claims: the Limited Expressivity Claim, the Inputted Expressivity Claim, and the Decoding Claim. We now reconsider each of these claims in light of our analysis of the LoL players’ reports about how they experience, and make use of, the expressive vitality of avatars.^8^

### The Limited Expressivity Claim Revisited

The Limited Expressivity Claim argues that avatars simply do not have a sophisticated enough expressive repertoire to allow us to perceive the other player’s thoughts and feelings through their avatar. When scrutinising the options that players have to express themselves in-game, focus is usually placed upon how players can: (i) design the appearance of their avatar; (ii) input pre-programmed ‘emote’ functions, such as waving, blowing a kiss, bowing, that their avatar performs; and (iii) use in-game chat functions to type and talk to one another over headsets. This often leads researchers to highlight the difference between encountering avatarial bodies in-game compared to face-to-face encounters in the real world.

Recalling the quote from Stern from earlier, we might suppose that due to the limited expressivity of avatars, in-game interactions are precisely experienced as “digital rather than analogic”—i.e., as lacking the aliveness and dynamism of human action. This quote may even suggest that interactions that take place on digital platforms are incomparable with the actions and movements of physical bodies. On the contrary, however, our analysis of the practitioners’ experiences lends support to the idea that vitality as a form of expressivity is present in these digital spaces. The practitioners describe how they experience the dynamic movements, actions and interactions of avatars as expressive. This expressivity is not found in discrete facial expressions or gestures but refers to the holistic style that a human-controlled avatar has. This motivates our claim that players perceive the *vitality* of avatars in-game.

The informants describe a rich array of expressive styles that human-controlled avatars have. Player-controlled avatars can move and act hesitantly to aggressively, calmly to erratically, frustratedly to nervously. Seeing an avatar moving around in a frenetic way, for instance, might reveal that the player is stressed out. The informants do not simply describe the human-controlled avatars as expressive but describe how this tells them something about the player ‘behind’ the avatar. Indeed, as the informants highlight, seeing the vital style with which another player’s avatar is moving gives them crucial information about how that player is doing, what emotional state they are in, what kind of player they are, and this informs how they engage with and interact with that particular player. Notably, players also describe how this expressivity of human-controlled avatars can dynamically shift over the course of a game as a player’s style moves, for instance, from calm to aggressive to stressed. Indeed, the vitality of another player not only seems to be perceived by other players but can emerge through on-going interactions *between* players.^9^ As players engage with one another, their expressive actions and movements reciprocally influence one another, leading to the interactive “dance” that Lyn describes.

Based on our analysis, the LoL players’ accounts arguably add further nuance to our understanding of vitality by emphasizing that, despite not resembling offline bodily interactions, human-controlled avatarial interactions can still have a wide range of vitality contours and thus be expressive in an analogue way even in digital contexts. In other words, the practitioners’ descriptions emphasize that what makes an interaction analogue rather than digital is not dependent on whether this interaction in fact takes place in a digital space, and, consequently, that Stern’s framework of vitality contours can help illuminate virtual interactions too.

Interestingly, the participants also report being able to easily differentiate between avatars that are bots and those that are controlled by other players. They describe the bots as not human-like, as being *almost* human but not quite. There are two ways in which we might interpret the participants’ descriptions of bots as ‘lacking humanity’ within the framework of vitality. On the one hand, we might conclude that the bots simply *lack* expressive vitality. Indeed, Stern precisely speculates that when interactions lack vitality, we would experience them as digital rather than analogic (Stern, 2010, 4). The way the participants describe the bots as not quite human-like may, then, be a real-world example of this very kind of experience. On the other hand, we might want to allow that bots still have some kind of vitality but a vitality that is essentially different to the vitality that humans and human-controlled avatars express.

Either way, the participants emphasise that there is something *distinct* about the way human-controlled avatars move and act. In contrast to bots, we might think that even though a player is mediated by their avatar, their teammates and opponents can still see their mind *in action*. As noted above, what seems suggestive is the way that the vitality of human-controlled avatars dynamically shifts in response to other players and the situation. This sensitivity and responsiveness to the situation in-game might be described as revealing a certain “liveliness” (Fuchs, 2022) that human-controlled avatars have. A liveliness that arises out of a socially and situationally sensitive awareness that informs and shapes how a player acts in game. This liveliness might be linked to an experience of the human-controlled avatars acting in an autonomous way that is qualitatively different to the automatic pre-programmed movements of a bot.

By paying attention to the dynamic, temporally extended way in which avatars move and interact in-game, we highlight vitality as a specific form of expressivity that is not typically considered by those who argue that avatars lack the kind of expressivity that human bodies have. Note that this does not fully undermine the Limited Expressivity Claim—one might be persuaded that avatars can express vitality, even that this gives us some social understanding of the player ‘behind’ the avatar, while maintaining that, compared to human bodies, avatars have a more constrained expressivity. Nevertheless, bringing vitality into the picture adds a kind of expressive possibility that has not yet been considered in the context of avatar bodies.

### The Inputted Expressivity Claim Revisited

The Inputted Expressivity Claim argues that, irrespective of the range of an avatar’s expressive repertoire, there is something fundamentally *different* about the expressivity of avatars compared to our physical bodies because any expressive behaviour that an avatar might enact must be deliberately and consciously inputted by the player. As such, avatars do not display the pre-reflective, unconscious body language that physical bodies do. There is, it is argued, a laboriousness about instilling an avatar with expressivity that is markedly different to our bodily expressivity.

While examples of directly inputting commands in order to signal expressivity in LoL, such as waving or laughing through one’s avatar, do exist, this is by no means the only way by which the in-game avatars can be expressive. As the practitioners’ descriptions show, avatarial vitality is not something that happens at the click of a button, but something that emerges through the temporally extended movements and interactions of the avatars. Indeed, this expressivity can and often does happen unintentionally during performance. Remember that Lyn specifically notes that it can be hard to mask one’s emotions in game, that the movements of one’s avatars betray what one is feeling. Given that showing one’s stress or nervousness in-game can be tactically problematic, as it reveals one’s (lack of) confidence, competence, and even spotlight oneself as a target for tilting, it can be presumed that often this vitality is precisely not something that the players are deliberately or consciously inputting into their avatars. Rather it is something that emerges through the *way* they are playing and engaging with each other.

Taking vitality as a mode of expression into account rebuts the claim that expressivity is something that must necessarily be cognitively, laboriously, or deliberately inputted by the player. Players might be commanding their avatars *what* to do (e.g., run to the other side of the map, attack an opponent, hide) but not *how* they do it (e.g., aggressively, nervously, hesitantly). Indeed, the vitality described by the informants often sounds like the kind of unconscious body language which it is suggested that avatars cannot possess.

In Section 6.3, though, we analyzed how players not only see the vitality of other players but also that they sometimes use and manipulate their own vitality style to pose as less confident or competent than they really are. A skilled player might mimic the nervous or hesitant movements of a less skilled player to hide their prowess. This suggests, then, that vitality can be inputted into the avatar deliberately by a player. While this may not be as cognitively onerous as scrolling through a menu and picking an ‘emote’ function, it does suggest that players can intentionally and deliberately *perform* vitality; that it is not always automatic and pre-reflective. At first glance, this appears to support the Inputted Expressivity Claim that the player must decide to input this expressivity into the avatar. However, just as using our physical bodies to perform expressive behaviour does not eliminate the possibility of pre-reflective bodily expressivity, the deliberate performance of vital styles with an avatar does not refute the occurrence of pre-reflective avatar vitality. It is also interesting to note that in order for the deception to work, the opponent players must perceive the nervous or hesitant vitality as giving them access to the player’s real emotional state and skill level. We might suppose, then, that the very success of performing vitality, rests on vitality typically being something that is not deliberately inputted by the player.

### The Decoding Claim Revisited

What, then, about the Decoding Claim? This is the claim that when we interact with people in the physical world, we do not need to engage in cognitively onerous processes of working out what bodily expressions mean but we see bodily expressivity as immediately meaningful and salient. In contrast, writers such as Dreyfus claim that when it comes to avatars, we have to explicitly decode any expressive signals given by avatars and work out what this might reveal about the player.

How the informants’ descriptions of perceiving and using the expressive movements of avatars relate to the Decoding Claim is not entirely clear. It is notable that a number of the informants at times describe being able to ‘read’ the body language of the avatars. This may sound like support for the idea that players must decode what the expressive movements of the avatar might reveal about the avatar’s player. However, it is also notable that the informants do not describe the avatars as signalling or performing vital styles. Rather they describe how they *see*, and are often surprised by, the human players’ emotions, intentions and skilfulness *in* the way their avatar performs and moves. We do not, then, find descriptions that suggest that the players must engage in some kind of cognitively onerous activity of ‘figuring out’ the expressive actions of the other avatars, nor descriptions of having to make inferences about what the other players are experiencing.

As such, the reports do not appear to suggest that the players are adopting a bifurcated approach of seeing a signal and then cognitively decoding it. Indeed, they report seeing the avatarial movements *as* expressions of the player. It is not that they perceive the avatar as signalling stress, rather, they seem to perceive the player as stressed. Indeed, in light of the participants’ descriptions, the claim that players must necessarily cognitively figure out what the other is doing just seems overly laborious—particularly when we consider the fast-pace at which players must perceive, adapt and respond to the expressivity of their opponents and teammates in the heat of battle.

It is here worth reflecting on Gallagher’s description of social perception as *smart.* In order for our social perception to become smart, experience and familiarity are important. We are not born with a pre-programmed understanding of all bodily expressivity. Our social perception is informed and shaped by our experience, it develops over time and (hopefully) becomes more sophisticated as we grow. We also talk of certain people being more socially sensitive and perceptive—able to pick up on subtle bodily cues and respond to them appropriately. Our social perception is also culturally informed and embedded (Andrada, 2019; Haslanger, 2019). Our social perception of nodding as immediately conveying agreement is smart in one culture but can lead us astray in others. In saying that social perception is *smart*, then, and that we often directly and immediately perceive the meaning of bodily expressions, is not to say that the smartness of our perception is fixed or static.

While the informants’ descriptions speak to a relatively rich and salient array of avatarial expressivity, they do so specifically in relation to the interactions of skilled LoL performers. In this regard, the informants’ ability to distinguish between a bot and a human-controlled avatar and to see play styles as having different vitality contours will likely differ from that a complete LoL novice. The ability to experience avatars as having vitality contours might, in this context, be described as a form of social ‘know-how.’ Notably, know-how, as typically conceptualised, is not understood as a set of beliefs, propositions, or representative knowledge that must be intellectually deployed by an agent (e.g., Di Paolo et al., 2018; Hutto, 2005; Myin & van den Herik, 2021; Rolla & Huffermann, 2021). Rather, ‘know-how’ is a practical knowledge that is developed and attained through practice and skilful interaction by an agent with their environment and others. That is to say, just as know-how can be seen as necessary for coming to perceive vitality in offline settings, so too know-how might be essential for coming to perceive vitality in LoL. This suggests a fruitful framework for perhaps unpacking why practitioners do not describe their experience of human-controlled avatars as expressive in terms of something that they must think about, infer, or puzzle out. Rather, it is part of the skillset acquired as a competent player familiar with the game. Developing this ability to see and use avatarial expressivity allows one to excel as a player and, as such, gaining this social know-how may be essential in order to play the game successfully at a certain level.

If, as one LoL practitioner notes, there is a correlation between skill level and the sophistication of a LoL practitioner’s social understanding (Interview Liam, lines 393, 400, 413–418, 420), the informants’ descriptions might not resonate with a novice gamer sitting down with LoL for the first time. To put it another way, the requisite know-how might be shared among certain communities, e.g., sufficiently experienced gamers, and not others. In this context, appreciating the saliency and relevance of vitality contours of avatarial interactions of LoL has been made possible precisely by turning to the perspectives of experienced, familiar informants. In other words, the LoL informants’ descriptions underscore the continuous importance of looking to actual, smart perceivers’ experiences of avatarial interactions when making claims about the limits of avatarial expressivity.

## Conclusion

Based on reports from expert LoL players, we have argued that discussions about the expressivity of avatars have overlooked ways in which the vitality contours of avatars can be perceived and used by skilled players and that perceiving vitality can give rise to social understanding of the player ‘behind’ the avatar. We have further argued that this evidence also suggests that vitality is not necessarily inputted into the avatarial body but emerges through gameplay, and that this vitality can be perceived in the movement of the avatar, rather than through onerous cognitive inference. Furthermore, the ability to perceive avatarial expressivity in this way, we have argued, will depend on the person’s history and familiarity with the relevant avatar movements and interactions.

Following our analytic strategy laid out in Section 5, our findings not only push back against critical claims found in contemporary literature on avatarial expressivity, they also open up novel and interesting research questions. One such question concerns where and how expressivity and social understanding between humans using avatars can emerge in the first place. With an eye especially towards avatarial *interactions*, we suspect the literature on participatory sense-making (De Jaegher & Di Paulo, 2007; Fuchs & De Jaegher, 2009; De Jaegher et al., 2017), as well as joint attention and action (Pacherie, 2012), might shed further light on this issue.

A second, related question concerns potential differences between being a player and being a spectator when it comes to seeing avatarial expressivity. More specifically, it remains an open question to what extent avatarial engagement and interaction impacts or is necessary for seeing other human-controlled avatars as expressive. With the rise of competitive, organized video gaming as spectator sports, what part, if any, does vitality play in the spectating experience of avatars competing? Based on the results of our analysis, we suspect that familiarity with the game will plausibly factor into this, too. While one informant did touch upon the difference between participating and spectating, we were unable to draw any final conclusions on this question.

Third, while we suspect vitality contours will play a role in games beyond LoL, as well as other avatar-based social platforms such as *Second Life* and *Gather Town*, their precise place in the myriad of different avatar interactions that exist today remains an open question. On the one hand, if a game like LoL, with its bird-eye perspective, lack of photorealistic avatars, and interactions often distinct from offline, physical forms of interaction, can still facilitate salient expressivity relating to other people’s mental states, it might well be that other forms of digital interaction with stronger similarity to human-like perspectives and traits can afford even more salient degrees of such expressivity. On the other hand, in the spirit of looking beyond avatars’ ability to mimic our physical bodies, we also find it particularly interesting to consider avatar-based platforms where interacting humans control not only a single, delineated avatar that they move around. Consider the fast-paced strategy game StarCraft II. Here, competing players are in control of entire military complexes, constantly undergoing construction and expansion, as well as vast clusters of armies moving in unison—all of which takes place at interactive rates much higher than LoL (Lowood, 2007). As noted, avatars come in numerous forms, with different aesthetics and ways in which players control and move them. Looking to these different forms of avatar interactions with vitality in mind, we suspect, will help us make better sense of the expressive capabilities of avatars and of ourselves.

## Data Availability

This is not applicable.

## Footnotes

1 The field of VR research more specifically has likewise explored avatar embodiment and similar phenomena (see, e.g., Peck et al., 2013; Slater et al., 1999).

2 We discuss Stern’s distinction between “analogic” and “digital” in greater detail in Section 7.1.

3 As noted in the introduction, for this reason, we leave out of our discussion the place and role of avatars facilitated by VR and similar technologies.

4 A few exceptions to this exist in the game where a player might temporarily control multiple avatars.

5 A’map’ in LoL is the term used for the entire, available virtual space itself. The players do also have access to a ‘minimap’ that indicates in more abstract terms what is happening on the map itself.

6 For an interesting discussion of how players control, and even couple with, non-human avatars, see Rolla et al. (2022).

7 Similar experiences are expressed by top-ranking players in games analogous to LoL, such as *Defense of the Ancients 2* (DOTA 2) where bot technology is more advanced. DOTA 2 star-player Danil ‘Dendi’ Ishutin, fighting (and losing to) an especially sophisticated AI-driven bot, was asked whether the highly capable bot felt “like a player, like a person”. To this, Dendi simply proclaimed “Uhm… Nope!”. Echoing Lyn’s description, Dendi would elaborate after the match that the bot “…feels a little bit like [a] human, but at the same time it’s something else” (Mashable, 2017).

8 For a discussion of vitality in the context of social media, see McCoster (2015) and for a discussion of vitality in the context of instant messaging, see Osler (2021, 2022).

9 For a general discussion of how individuals experience ‘betweenness’ in digital spaces, see Osler & Krueger, 2022.
